# Diffusion of community heart failure service innovation in Northamptonshire, England: a qualitative study

**DOI:** 10.1017/S1463423625000301

**Published:** 2025-04-24

**Authors:** Taliha Samar, Gupteswar Patel, Aloysius Niroshan Siriwardena

**Affiliations:** 1 3^rd^ year medical science student, Lincoln Medical School, Universities of Lincoln and Nottingham, UK; 2 Post Doctoral Research Associate, Community and Health Research Unit, School of Health and Social Care, University of Lincoln, Lincoln, UK; 3 Professor of Primary and Prehospital Care, Community and Health Research Unit, School of Health and Social Care, University of Lincoln, Lincoln, UK

**Keywords:** Community cardiology, community engagement, Diffusion of innovation, Northamptonshire

## Abstract

**Introduction:**

Heart failure is a complex disorder, that can require hospitalization and specialist care, which patients may experience challenges accessing. In Northamptonshire, an innovative approach to heart failure services was introduced to address these challenges. This study aimed to explore and understand the diffusion dynamics of the heart failure service in Northamptonshire, focusing on adoption and implementation determinants.

**Methods:**

This qualitative study involved 11 in-depth interviews with four patients, two community carers, one general practitioner, one nurse, one programme director, and two interviews with a community cardiologist. The diffusion of innovation-guided inductive and deductive thematic analyses were used to identify themes and subthemes.

**Results:**

The community heart failure services incorporated community cardiology clinics and community asset groups. Implementation of these innovations was characterized by competent leadership, positive managerial relationships between community cardiologists, general practitioners, and third-sector professionals, a ‘tension for change’ to reduce hospital admissions, improve access, and dedicated funding (‘slack resources’). The ‘relative advantage’ identified by both service providers and patients was access to specialist care closer to home, rehabilitation, education, and nutrition services. The heart failure innovation aligned with the organizational values of primary care and third-sector organizations, facilitating readiness for adoption and implementation. Challenges emerged from limited management accountabilities, such as inadequate administrative and information technology support, hindering the implementation.

**Conclusion:**

The heart failure innovation was perceived to improve care, navigating both facilitators and challenges. The diffusion of innovation theory highlighted the importance of governance and the performance of community heart failure services within a complex intervention context.

## Introduction

Empirical evidence on the diffusion of innovations is crucial for understanding the implementation dynamics of health service interventions in real-world contexts (Dearing, 2009). In Greenhalgh et al’s terms, the introduction of patient and public “Community Asset Groups” (CAGs) and specialist “Community Cardiology Clinics” (CCCs) in Northamptonshire, England were innovations introduced to improve Heart Failure (HF) services. These innovations were implemented by a community cardiologist, general practitioners, and community carers from third-sector organizations.

HF is on the rise, affecting 64 million globally in 2017 (Shahim *et al.*, 2023). The prevalence in the UK was 920,000 in 2018, with 200,000 additional diagnoses annually expected (Rough, Adcock and Baker, 2021). An aging population and rising life expectancy increase prevalence (Marani *et al.*, 2020; Remawi, Gadoud and Preston, 2023). HF accounts for 5% of UK adult emergency hospital admissions (Cleland *et al.*, 2012; Whyte *et al.*, 2014; Braunwald, 2015). England had 94,185 HF admissions in 2020 (BHF, 2020), with a length of stay twice the average of other conditions (11-days vs. 5) and a 16% readmission rate within 30 days of discharge (Simmonds *et al.*, 2015). Diagnosing HF in co-morbid patients is particularly challenging due to complex symptoms and the risk of misdiagnosis, which often requires specialist involvement that is frequently inaccessible in rural areas. There are inequities in access to diagnosis at the primary and community care levels that may lead to unnecessary emergency department referrals (MacKenzie *et al.*, 2010).

The management of HF at primary, secondary, and community levels are essential. Research on local health systems providing HF care emphasizes the need for more integrated and coordinated services to deliver patient-centred care, considering the complexities in conditions and inequality in access to care (Hayes *et al.*, 2015 and Muth *et al.*, 2019).

### HF care intervention in Northamptonshire

The community cardiologist started working in March 2022, and three CCCs and two CAGs were functioning when the data collection of this study commenced in September 2022. The initiative was funded through Integrated Care across Northamptonshire (ICAN) and a third-party company for a pilot period of 12 months.

The implementation of the HF care intervention was led by the community cardiologist, and involved collaboration with general practitioners (GPs) to establish CCCs within primary care settings, providing specialized HF services and bringing specialist care closer to patients’ homes. The three CCCs operated within separate general practices in different regions of the county. The first CCC, located in the county’s western region, had 53 patients with heart failure registered. Its multidisciplinary team included general practitioners, advanced clinical practitioners, nurses, a pharmacist, a physiotherapist, a social prescriber, a mental health worker, and health and wellbeing coaches. The second CCC was also situated in the western region, with 136 registered heart failure patients. This practice comprised general practitioners, nurse practitioners, nurses, healthcare assistants, a clinical pharmacist, a physiotherapist, and administrative staff. The third CCC, located in the county’s northern region, had 43 registered heart failure patients and employed general practitioners, a podiatrist, nurse practitioners, nurses, a clinical pharmacist, a community nurse, healthcare assistants, and administrative staff (Department of Health & Social Care, n.d.). The community cardiologist joined the general practice teams to form and function the CCCs.

**Figure 1.**
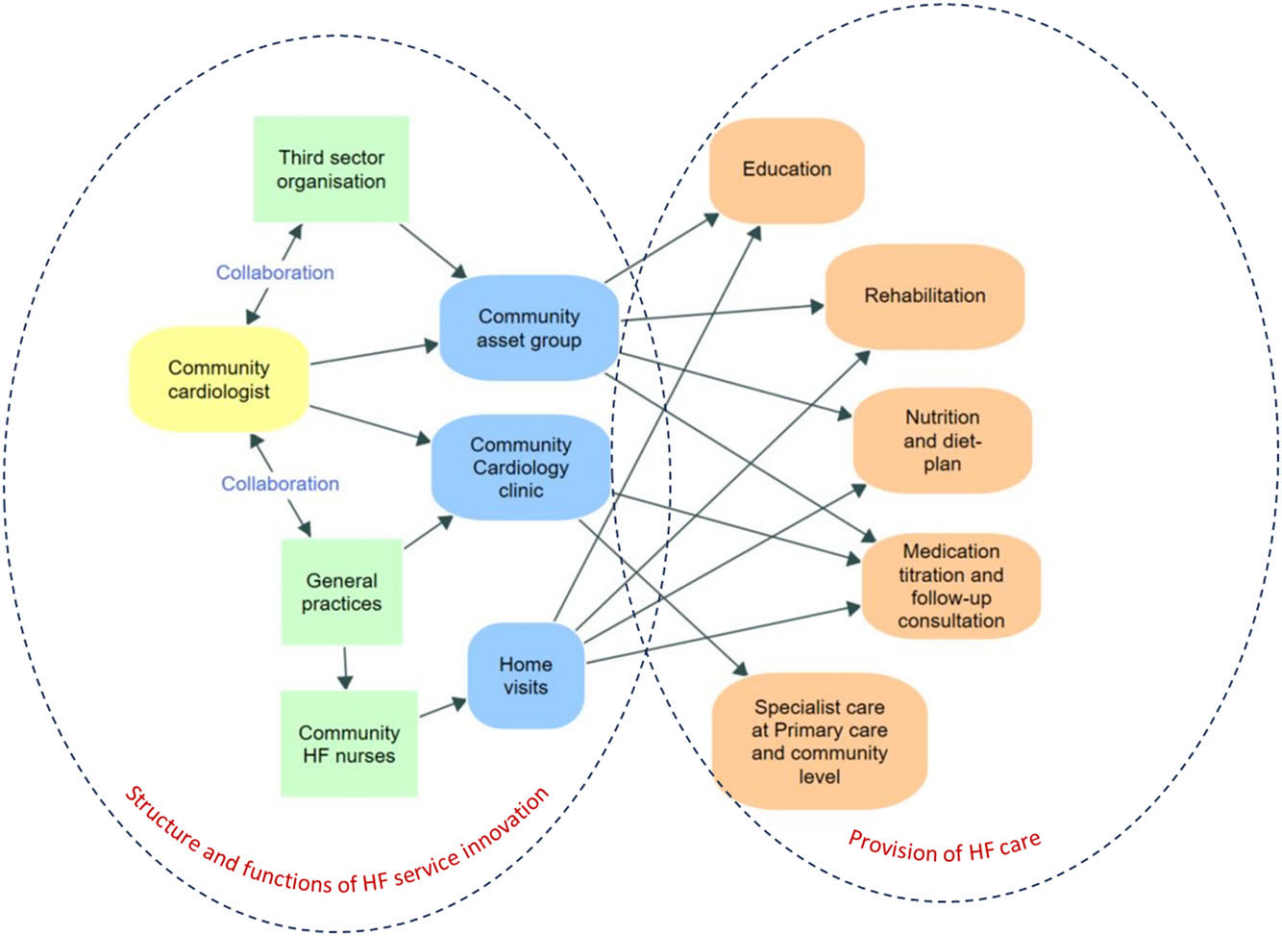
Northamptonshire innovative HF care model.

Concurrently, the community cardiologist collaborated with third-sector organizations to establish CAGs, also known as the ‘Pumped-up’ group. Both CAGs were located in the western region of the county. One covered an area with 267 HF patients, while the other, located about 20 miles away, covered an area with 547 HF patients (Department of Health & Social Care, n.d.). The third-sector organizations included physiotherapists, rehabilitation coaches, social workers, nutritionists, managers, and administrators. The community cardiologist joined the third-sector organizations to organize and function the CAGs. These groups met fortnightly to provide education, rehabilitation, nutrition guidance, lunch, and social activities, with access to the community cardiologist and educators. Figure 1 illustrates various aspects of the HF care innovation.

### Conceptualization of diffusion of HF service innovation

**Figure 2.**
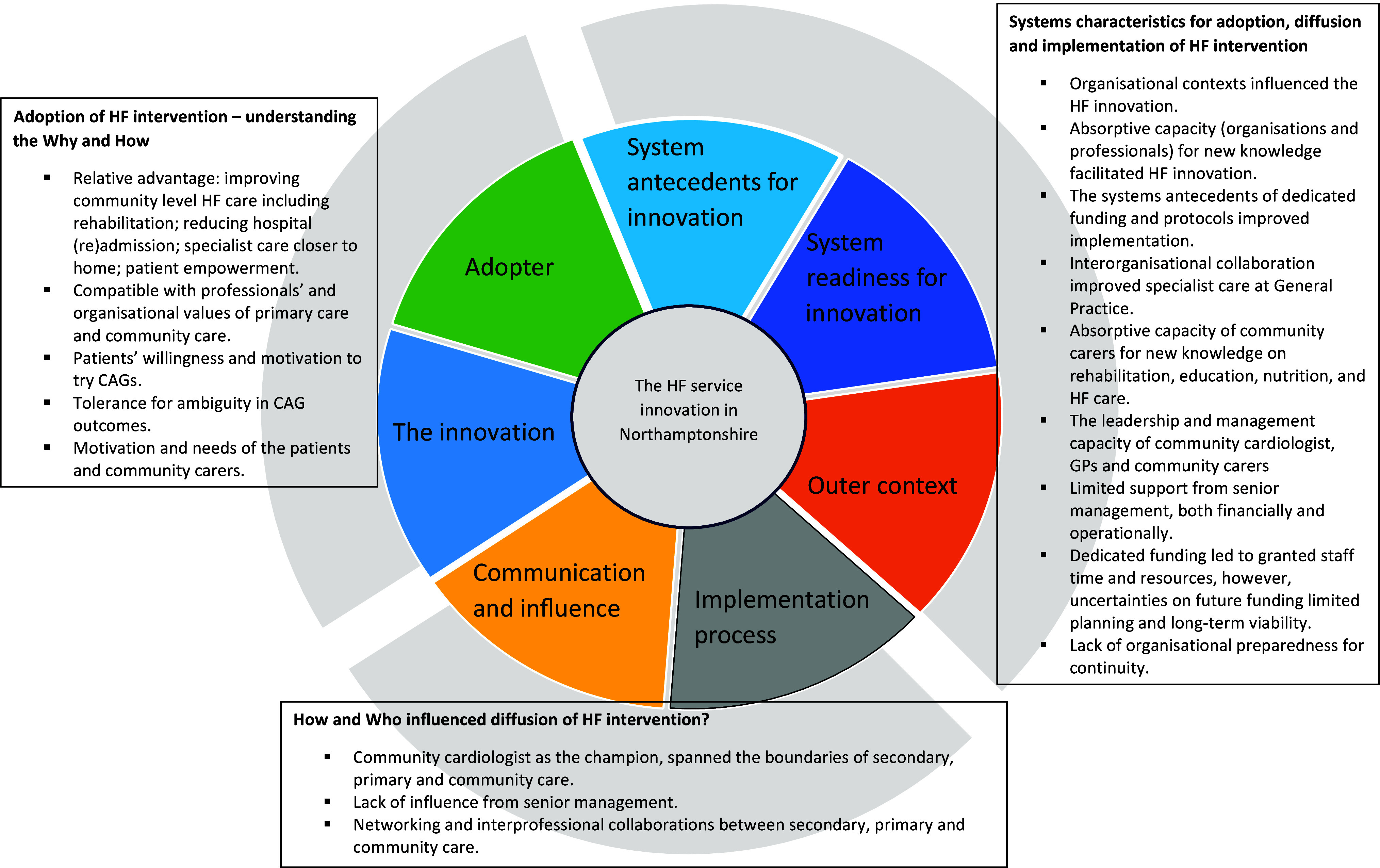
Thematic map of diffusion of HF innovation in Northamptonshire.

As HF services were newly introduced, diffusion of innovation theory was instrumental in understanding their implementation and evolution. The diffusion of innovation theory by Greenhalgh *et al.* (2004) includes various attributes and sub-attributes that influence the adoption and implementation of intervention (Figure 2). We have highlighted those relevant to our study.

According to Greenhalgh et al., an *innovation* is more likely to be adopted if it offers a relative advantage, aligns with the values of the adopting organization or team, and its benefits are observable. Individual adoption is crucial as well; motivated individuals who perceive the innovation as meeting their needs and goals are more likely to embrace it. This active *adoption* process aligns with their values, and feedback from intended adopters can refine the innovation for greater success.

*Communication and influence* play significant roles, particularly involving opinion leaders, champions, and boundary-spanners who introduce new ideas and strategies. Adoption is more likely when individuals share similarities—or exhibit homophily—with those advocating the innovation. *System antecedents* such as structural determinants, absorptive capacity for new knowledge, and a receptive context for change also enhance the likelihood of successful assimilation.

*System readiness for innovation* is crucial, allowing an organization to formally implement the innovation when there is tension for change and dedicated time and resources. The *implementation process*, following the adoption decision, is strongly dependent on system readiness and involves a nonlinear shift from implementation to routinization, often facing mid-implementation challenges. Key components for successful routinization include the autonomy of frontline teams, support from higher managerial roles, and effective intra-organizational communication.

Based on the diffusion of innovation theory, this study conceptualized the adoption and implementation of the HF service intervention in Northamptonshire. The application of this theoretical model enabled the interpretation and understanding of factors influencing the adoption of the intervention.

Several studies have investigated the diffusion of various health service innovations in other countries including the UK (McMullen *et al.*, 2015), the US (Sabus and Spake, 2016), Canada (Makowsky *et al.*, 2013; Munro *et al.*, 2021), Germany (Merkel *et al.*, 2015), and Singapore (Lim *et al.*, 2021). For example, McMullen *et al.* (2015) investigated the impact of rapid HIV tests on mean CD4 cells at the time of diagnosis in London. By employing the diffusion of innovation model, they identified characteristics of leadership, readiness for change, and a culture of capacity building for healthcare staff that influenced the implementation (McMullen *et al.*, 2015). This study applied the diffusion of innovation theory to explore the current state of HF care practices and understand how the diffusion of innovation model relates to explaining implementation dynamics, and explore and identify the key determinants influencing the adoption and implementation of the HF service intervention in Northamptonshire, with an emphasis on the role of health and social care professionals, organizational factors, and collaborations.

## Methods

### Research design and settings

This qualitative study was carried out in Northamptonshire, an East Midlands County of England, wherein the new model of HF services had been implemented.

### Data collection and participants

Using purposive sampling, we (GP and a Research Assistant) sent participant information statements and consent forms through e-mail to stakeholders involved in the HF intervention, and HF patients who had used services through CCCs and CAGs. Participants were provided a minimum of seven days to consider their participation and provide informed consent. Only those who provided written informed consent were recruited, ensuring voluntary participation. GP – PD Research Associate and a Research Assistant conducted (online) in-depth interviews with 10 participants (4 male and 6 female), including 4 patients, 2 community carers from third-sector organizations, 1 general practitioner, 1 community heart failure nurse, and 1 director from NHS secondary care, and 2 interviews with the community cardiologist. The interviews guided by tailored interview schedules based on participants’ roles, aimed to incorporate a wide range of experiences and practices. The data collection took place between October 2022 and February 2023. The interviews were audio-recorded with consent, transcribed, and lasted between 27 and 85 minutes.

### Ethics

Ethics approval was obtained from the Human Research Ethics Committee, University of Lincoln, UK (approval no. 2022_9784).

### Data analysis

We adopted a combination of inductive and deductive thematic analysis using Braun and Clarke’s thematic analysis framework (Braun and Clarke, 2013), supported by NVivo14 software for data management, coding, and analysis.

We (TS and GP) uploaded transcripts into NVivo14 and created nodes and sub-nodes in the software, and organized and synthesized the data by labelling portion of text or sentences with a short code, followed by grouping the coded texts. The open coding of 11 transcripts created a large number of codes, with newly identified codes systematically incorporated into the coding lists. We then revised the coding frame deductively, based on the broader diffusion of innovation-based attributes. We read and re-read the data outputs as they were presented for each code to identify the higher-level descriptive themes, and developed the subsequent interpretation using diffusion of innovation theory.

For example, the theme “relative advantage” of the innovation was developed through a structured process of thematic analysis. We began by carefully reading the interview transcripts to identify recurring ideas. Key points included the service providers’ emphasis on how the intervention improved patient management at the community level, reducing the burden on secondary care. Similarly, patients highlighted the convenience of accessing HF services locally.

Using NVivo, we developed codes to capture these reoccurring ideas: service providers focused on improved patient management and the reduced burden on secondary care, while patients highlighted access to care, convenience, and proximity within the community.

These codes, “the benefits to the patients and public” and “benefits to NHS”, were combined into the broader theme of “relative advantage,” reflecting how the HF intervention offered advantages compared to previous models.

## Results

Several broad diffusion of innovation constructs were recognized as important for understanding community HF intervention. The results are presented in three primary themes, outlined as subheadings below.

### Adoption of HF intervention – understanding the why and how

Several diffusion of innovation concepts illustrated the empirical and conceptual understanding of the adoption of HF interventions, including observable relative advantage, compatibility, context specificity, and the motivation of involved stakeholders.

#### Innovation factors

The community cardiologist highlighted the relative advantage of the intervention in improving patient management at the community level, thereby reducing the burden of hospital (re)admissions from secondary care. Furthermore, the new HF services as a strategic mechanism for broadening access to HF care, contributed to addressing prevailing inequity in HF services. Similarly, patients identified and observed the relative advantage of new HF services delivered through CAGs. Patients perceived accessing HF services within the community as a substantial benefit compared to the previous model, where visits to secondary care hospitals were necessary, in contexts with limited public transportation. Patients also highlighted the provision of care closer to home as another relative advantage.

*So you have got the Pumped Up! group there, offering that continuity [of care]. I think that’s sounded as though that’s been really key to your rehabilitation really…* (Patient_2)

The improved access to community cardiologists in community and primary care settings was another aspect of perceived relative advantage. The opportunity to consult with HF professionals within familiar community settings, health education, access to rehabilitation services, psychological support, and dedicated facilities to socialize were recognized as a relative advantage by patients. Considering these observed relative advantages, patients demonstrated an inclination to adopt innovative HF services. This increased readiness for adoption was a manifestation of the crucial role that perceived relative advantage and observability have in influencing patients’ attitudes and involvement towards the adoption of the new HF care model.

*You get to meet Dr [community cardiologist] and ask questions, even the educational bit…about a different aspect of heart health and diets and all sorts, there’s so much to learn.”* (Patient_2)

GP also expressed a relative advantage over the CCCs. The presence of the community cardiologist in the primary care facilities meant HF patients were also managed by specialists, reducing GPs’ workload and possibly reducing hospital admissions.

*“So, when we agreed that [community cardiologist] could have a room, [community cardiologist] came to meet us and discussed with us what they wanted to do, and it was all fine… It was just like going to see a GP, but seeing a specialist at the same time.”* (GP_09)

Compatibility was identified as a significant determinant in the adoption of innovations, being compatible with the values of individual professionals and participating organizations. For the community cardiologist and community carers, the innovations were aligned with the goal of promoting patient-led care in the community, and facilitating patient empowerment through health education. This alignment aimed to enhance patients’ ability to self-monitor their condition, thus reducing the likelihood of deterioration and preventing unplanned admissions.

*“We’re trying to make expert patients and trying to give them that information before people end up in crisis.” (Community-Carer_03)*

The CCCs also aligned with the values and norms of participating primary care organizations, integrating CCCs into existing primary care practices.

*“So, [community cardiologist] was seeing our patients in the clinic, as well as other practices’ patients, which wasn’t a problem. And at the same time, [community cardiologist] said to us if we had a patient with any other cardiological problem that we wanted to discuss as well, feel free to, which we did.” (GP_09)*

The intervention was compatible with ICAN and third-sector organizational values by undertaking care services in the community and addressing health inequalities. The CAGs incorporated education and opportunities for the patient’s family members to learn about HF and care approaches at home.

*“There are health inequalities for pretty much everything that we’re supporting but it’s how we address those. I think that we are making sure that everyone has access to the right information at the right times.” (Community-Carer_06)*

*“…it’s about managing a condition or a home environment so it’s the whole person… for example, yes we’re supporting the person that has HF but we’re also supporting their wider family and carers as well.” (Community-Carer_06)*

#### Adoption characteristics

Innovation adoption by the intended adopters was an important factor, relying on adoptors’ willingness to try and apply them, motivation, and tolerance for ambiguity. Motivation and propensity to try out the CAGs by the HF patients were important for adoption.

It was identified that individual traits, particularly *tolerance for ambiguity*, played an important role in the initial adoption of HF services and ultimately contributed to the successful adoption. Community cardiologists noted that, at the beginning of the intervention, some patients expressed less motivation to engage in CAGs, but later participated, demonstrating a considerable tolerance for ambiguity regarding participation in such new groups. These patients did not initially express a specific intention to join CAGs, and they expressed scepticism regarding the usefulness of these groups in meeting their needs or goals of recovering from HF. A prevailing patients perception was the concern of ‘what’s in it for me’. Despite patients’ initial uncertainty, they ventured into innovative HF services. Upon active involvement in the CAG sessions, patients understood that their requirements related to HF management and recovery were sufficiently discussed and addressed. Community cardiologist shared that many patients transitioned into becoming regular members of the CAGs with attendance at each session. Therefore, individual attributes of trying out and using intervention were vital in the successful adoption of HF innovation.

*“Oh, I really don’t think it’ll be my thing, but I’ll come and support it for a couple of sessions and that’ll be it, but I don’t expect to do it long term.” He’s never missed a session, and we’ve had quite a few people like that* (Community cardiologist_10)

#### Motivation and needs of the patients and community carers

Patients were motivated by an optimism to recover from HF and sought individualized care. Patients often expressed frustration with insufficient support at primary care. The implementation of the new HF intervention, access to a community cardiologist in the CCCs, and the private consultation in the CAGs improved and maintained patients’ motivation to participate in the intervention.

*“…they’re a GP. They haven’t got the time to sit and talk to you individually…”* (Patient_04)

*“…has a room set aside there, so we can book a one-to-one [consultation] with [community-cardiologist]…”* (Patient_04)

Community carers with collective motivation and requisite capacity demonstrated a desire to adopt and implement the HF intervention with the objective of providing community-level HF care. This professional commitment aligned with CAGs, where the third-sector organizations’ role was to receive a patient referral from secondary care, and their enrolment in CAGs. In effect, the delivery of HF care through CAGs enabled individual community carers and third-sector organizations to adhere to their values.

*“With Pumped Up! it’ll be different because we want to have those referrals in the right way because they meet that criteria.”* (*Community-Carer*_06)

#### Concerns and future recommendations by patients

The patient enrolment based on recommendations from existing patients was established as a facilitative mechanism, enabling a more inclusive dissemination and adoption of the intervention. HF patients observed that the CAGs were inaccessible for some patients. This limitation arose from referral-based patient enrolment, thereby hindering the broader reach of CAG sessions, which limited the adoption and contributed to perpetuating inequality in access to HF care. Recognizing these concerns from patients, the community cardiologist and carers urged enrolled patients to encourage other HF patients in their community to participate in the CAG sessions.

*“Obviously I know you can’t access them unless you are referred, you’ve got a referral, so that’s… to see that you can see other people that need them, and it was only on a referral basis at that moment.”* (Patient_02)

### How and Who influenced the diffusion of HF intervention?

The communication and influences that spread the HF services were primarily led by the community cardiologist, who acted as a ‘champion and boundary spanner’ while working across all levels of care to implement the intervention. The patients also influenced the diffusion by actively disseminating CAGs with their friends and family to spread awareness.

The community cardiologist, as a change agent, established the community HF services. Their leadership skills and clinical knowledge contributed to the complex processes of planning and implementing various aspects of the CCCs and CAGs. The study participants uniformly expressed admiration for the community cardiologist’s creative initiative and improvements to HF services in the communities.

*“…because they’ve got a great cardiology doctor there.”* (Patient_05)

However, the diffusion of the HF innovation faced challenges enforced by a lack of *influence* from ICAN decision-makers, and responsible intra- and inter-organization communication. This limitation manifested when the community cardiologist faced challenges with IT access in community settings. Lack of support from these leads hindered the community cardiologist’s efforts and resulted in a lack of administrative assistance for the functioning of community HF services, which has likely contributed to a deceleration in the diffusion of HF services.

*“We know there have been a lot of deficiencies from lack of admin and secretarial support, but the IT at the hospital has been a real hindrance in terms of supporting the HF development.”* (Community cardiologist_10)

The community cardiologist acted as a boundary spanner and integrated new HF intervention between secondary, primary and community care through the CCCs and CAGs.

*“I know that [cardiologist] is seeing patients within the community… the Pumped-Up groups.”* (Director-Cardiology_07)

The community cardiologist initiated the intervention by identifying, networking, and collaborating with primary care and third-sector organizations. Their active involvement enabled collaborating organizations and professionals to understand, recognize, and adopt the new model of HF care delivery with their respective organizations, facilitating the delivery of specialized care close to patients’ home and integration of care across different health system tiers.

*Had it not been for [community cardiologist] dedication to doing that, because [cardiologist] spent many hours of own time supporting collaborators. I think you just need somebody that is flexible. You need somebody that actually understands how charities work, secondary care works as well as primary care because GPs work in a very different way…”*(Director-Cardiology_07)

*Homophily* amongst patients was identified as important in peer support and advice. The CAG-enrolled patients shared a similarity with other patients in experiencing HF symptoms. These patients desired to recover from HF by using services available at the CAGs, and learning from other HF patients resulted in the emergence of a peer support network. This interplay of shared narratives provided mutual understanding and learning opportunities for other patients undergoing similar challenges. Thus, patients collectively diffused new HF services, facilitated inclusivity and homophily, and increased the likelihood of adopting the services.

*“So it’s a social interaction with other people who- you’ve now got something in common, you’re all sharing the same heart condition, learning from one another”* (Patient_2)

### Systems characteristics for adoption, diffusion, and implementation of HF intervention

Health systems and organizational contexts influence the success of innovations, affecting the adoption and incorporation of new ideas into day-to-day professional activities (Greenhalgh *et al.*, 2004). System antecedents for innovation are influenced by structural determinants and absorptive capacity (organizations and professionals) for new knowledge.

The systems antecedent was illustrated in the context of the ICAN, participating primary care and third-sector organizations, demonstrating functional differentiation attributed to their commitment to implementing the HF intervention. Particularly, ICAN secured funding and developed protocols for the new HF care model, aligning with its organizational structure. Similarly, the intervention also aligned with the structural and functional aspects of primary care and third-sector organizations, providing dedicated facilities for CCCs and CAGs, respectively.

*“…was funded externally from iCAN…, and that was specifically to set up some community heart groups for HF.”* (Community cardiologist_10)

Primary care organizations participating in CCCs provided space to the community cardiologist and incorporated their specialist care. Concurrently, community carers from third-sector organizations adapted to the CAGs, which involved new knowledge on rehabilitation, education, nutrition, and the recovery process. Thus, the *absorptive capacity* of participating organizations and professionals facilitated the adoption and implementation of the HF intervention.

*“…my own personal development and learning about these things for my [new]role… I learn a lot from [community cardiologist], from listening to talks.”* (Community-carer_03)

The *leadership and management capacity of key individuals* affected the HF service implementation. The community cardiologist, ICAN, and third-sector organizations’ professionals worked together in implementing the HF intervention and attempted to address emerging challenges with varying outcomes.

*“But, from that, myself and [community cardiologist] worked together and planned the Pumped Up! groups.”* (Community-carer_03)

The leadership and management capacity of community cardiologists was exemplified in establishing CCCs, especially in circumstances where setting up CCCs in primary care was difficult, with some primary care organizations demanding payment for primary care facility use. However, the community cardiologist was able to collaborate with other primary care organizations, to access their facilities without incurring any costs but in return seeing their HF patients and delivering specialized care at the primary care level. These influential managerial relations promoted a receptive context for change in adopting HF intervention.

*“…you form working relationships with people. Obviously, they were giving us their space without any cost to the Trust or the service, so we got that space for free essentially. But in return for that, they got a [community] cardiologist that was able to support and advise them when I was there, and I think they got a lot of benefit out of that.”* (Community cardiologist_10)

Moreover, senior management support was important in making decisions that determined the intervention’s financial and implementation mechanisms. There was a limitation in obtaining direct support from the senior management of the NGH and ICAN, both financially and operationally, thus hindering the implementation process. As a result, the community cardiologist was working without administrative support at the community level and lacked access to institutional support and governance, hindering their readiness to function efficiently and implement the intervention as planned.

*“If you don’t have that support higher up, it’s really hard to take things through effectively because you need that drive from people at the top of the management chain to drive it through. And unfortunately, there just wasn’t that at all.”* (Community cardiologist_10)

The increasing hospitalization rates and inaccessible care at the community level led to *tension for change* within the NGH. In response to this tension, the HF intervention was devised and implemented as a transformative solution and was expected to improve access to HF care, mitigate the frequency of hospital (re)admission, and introduce rehabilitative services at the community level–previously unavailable before intervention.

With dedicated funding, the HF care implementation was facilitated by the allocation of dedicated staff time and resources. However, staff attrition within ICAN administration created complexities in the utilization of available funding. There were also IT-related challenges for the community cardiologist to practice at CCCs, which led to administrative burden in patient consultation.

*“We haven’t decided yet how I’ll share any information with the hospital, but it’ll be more efficient being straight into SystmOne [a digital patient record system in NHS] and sorting out the prescriptions locally. That has been a challenge of working differently and that was a new way of working for the Trust,”* (Community cardiologist_10)

Although the HF intervention was initiated with dedicated funding as part of the pilot intervention, future funding was not assured. Therefore, continued activity remained uncertain, which posed challenges for planning and the long-term viability of the HF intervention beyond the initial funded phase, highlighting a lack of organizational preparedness for continuity.

Furthermore, the implementation of the intervention depended on the degree of autonomy possessed by key professionals involved. For instance, the community cardiologist acquired autonomy over their professional practice and day-to-day activities within the scope of community HF services. This extended to the selection of venues for CAGs, coordination with primary care organizations, provision of training to community carers for support, facilitation of operational challenges, participation in regular staff meetings, organization of CAG sessions, and delivery of health education lecturers to patients. Similarly, community carers gained autonomy in selecting the processes of delivering rehabilitation, dietary guidance, exercise education, and access to the gym for patients. This autonomy and operational decision-making of these key individuals operating at the community level were posited as mechanisms that increase the likelihood of successful implementation.

## Discussion

The exploration of the diffusion of this HF care intervention revealed a dynamic healthcare context with a combination of secondary care, primary care, and community care. This study delineated an understanding of the complex mechanisms governing the attributes of innovation and influencing widespread adoption and implementation. This study also deconstructs the diffusion dynamics by analyzing the process of implementation, highlighting system characteristics, and addressing questions of why, how, and by whom adoption, diffusion, and implementation were facilitated or hindered. Health and social care organizations that established governance of intervention implementation and provided leadership support tend to have higher adoption of innovative care practices. Figure 2 shows the key themes identified that reflect how HF care innovation is influenced, adopted, and diffused in Northamptonshire.

Complex interactions of health and social care professionals, organizational values and governance mechanisms, and collaboration shaped implementation. In line with the diffusion of innovation framework (Greenhalgh *et al.*, 2004), it was evident that the relative advantage of improved accessibility to HF care within the community, patient-centred services, and workload reduction from GPs contributed to the adoption and readiness of community cardiology innovation. The intervention was well-aligned with the values and objectives of healthcare professionals, primary care organizations, ICAN, and third-sector institutions to improve HF services. Patients, GPs, and community carers identified the HF model as simple and easy to adopt. However, there were challenges in the functioning of community HF services, including a lack of administrative support, funding, and implementation. The dynamic interaction between these professionals and organizations allowed for adjustments and flexibility, contributing to the successful adoption and implementation. Thus, the empirical findings of this study are in line with Greenhalgh *et al.* (2004), and it is argued that professionals play an important role in the adoption of intervention, and professionals who are well-informed, motivated, and engaged in continuous learning with absorptive capacity for new knowledge are more likely to adopt and successfully implement innovative approaches.

Within the context of community HF services, the community cardiologist established an influential role, functioning as a champion and change agent. Despite these concerted efforts, challenges emerged from the lack of influence resulting from other transformational leaders within ICAN, hindering the implementation progress of community cardiology services. The community cardiologist, as a boundary spanner, attempted to integrate the spheres of secondary care, primary care, and community care in HF services. In line with the concept of interconnectedness, the attributes of innovation that affected functions of the HF intervention were not necessarily exclusive, and each attribute could influence other attributes and/or correlate with other factors (Espinosa-González *et al.*, 2019).

This study is the first of its kind to analyse the diffusion of a new and distinctive HF care model being implemented in Northamptonshire. This study makes an original contribution to knowledge by illustrating the availability of dedicated funding for innovation, the significance of primary care and third-sector organizations, their respective professionals, and patient’s participation in the implementation of HF intervention. Despite being led by specialist medical practitioners, implementation instances were dependent on the collaborative efforts of the aforementioned entities.

Available evidence from broader research on the diffusion of innovation and implementation science has illustrated comparable findings (Harting *et al.*, 2005; McMullen *et al.*, 2015; Darling *et al.*, 2021). These similarities reinforce the validity and applicability of the findings, providing confirmation to the notion that the diffusion dynamics identified in this study extend beyond the specific contextual factors of Northamptonshire and may translate to the dynamics of health services and HF innovation in similar contexts. Harting *et al.* (2005) identified that the integration of counselling services for patients showed higher adoption within outpatient cardiology clinics in the Netherlands. This adoption was associated with the role of “change-agent”, the non-complex innovation, and its compatibility with the organisational routines of cardiology clinics (Harting *et al.*, 2005).

Darling *et al.* (2021) used Greenhalgh *et al.* (2004) theory to investigate Canada’s first alongside midwifery unit and describe many factors affecting implementation, including funding and midwifery leadership skills. Similarly, this research also identified ongoing financial support, the role of champions, and the relative advantage of the intervention that influenced HF service implementation. By adopting the Greenhalgh *et al.* (2004) theory, McMullen and colleagues identified that the spread of an HIV diagnosis intervention in London exhibited distinct features, including effective leadership, positive managerial relationships, disposition towards change, staff training, and sufficient staff time (slack resources) (McMullen *et al.*, 2015). Similarly, this study also identified leadership and management capacity at multiple strata, positive managerial relationships, tension for change in reducing HF (re)admission, and improving community HF care influenced implementation. The dedicated funding facilitated the provision of sufficient staff time (slack resources), and the observability of perceived benefits by both frontline care professionals and patients.

### Implications for future practice

This study highlighted the need to strengthen transformational leadership within ICAN and NGH Trust. Decision- and policy-makers should prioritize the operational challenges, agreed accountabilities, and allocate support for the community cardiologist for improved implementation.

Decision-makers should prioritize resources for overcoming IT-related challenges to HF services, which may support implementation. Sustainable funding mechanisms are crucial for successful implementation and integration. Key organizations should devise long-term plans for sustainability, addressing funding uncertainties and organizational readiness. This will ensure that the improvements made in HF care delivery are maintained and continued in the future.

## Strengths and limitations

This study highlights the significance of recognizing, learning, and implementing similar future interventions. It also contributes to the theoretical discourses on the diffusion of innovation and health service implementation by offering empirical evidence.

Various participants with varied professional competence and personally lived experiences were interviewed to obtain an in-depth understanding of the intervention, mechanisms of diffusion, and organizational adoption. In-depth interviews gathered accounts of services that worked well and perceived recommendations. The absence of policymaker participation in the interviews may have limited the health system’s perspective on current and future funding. The small number of interviews in this study is a limitation in terms of the generalisability of the findings. However, as this is a qualitative study, the objective was to explore and understand participants’ experiences and perspectives on the new HF innovation implementation. The data collected enabled us to identify and understand the complexities of the innovation’s diffusion dynamics.

Future research should employ quantitative methods to analyse the impacts of the intervention on HF (re)admissions, mortality, and discharge timelines.

## Conclusion

The diffusion of innovation model was used to explore diffusion dynamics of HF services in Northamptonshire and identify key determinants of adoption and implementation. Several factors facilitated successful implementation, including dedicated and ongoing funding, effective leadership skills from champions, and efficient collaboration among secondary, primary, and community care. Factors that hindered implementation, requiring future consideration for efficient adoption and diffusion of the HF intervention included support from higher managerial roles, dedicated resources for sustainability, and interorganizational networks. In settings where new community HF services are much needed, the factors outlined in this study can be used to inform an intentional, strategic approach to implement this intervention to improve HF care. The integration and collaboration of health and social care professionals were significant in the diffusion, dissemination, and implementation of the HF intervention and beyond.

## Data Availability

The data that support the findings of this study are available on request from the corresponding author. The data are not publicly available due to privacy or ethical restrictions.
